# Ion-Pair Interaction and Hydrogen Bonds as Main Features of Protein Thermostability in Mutated T1 Recombinant Lipase Originating from *Geobacillus* *zalihae*

**DOI:** 10.3390/molecules25153430

**Published:** 2020-07-28

**Authors:** Siti Nor Hasmah Ishak, Nor Hafizah Ahmad Kamarudin, Mohd Shukuri Mohamad Ali, Adam Thean Chor Leow, Raja Noor Zaliha Raja Abd. Rahman

**Affiliations:** 1 Enzyme and Microbial Technology Research Centre, Faculty of Biotechnology and Biomolecular Sciences, Universiti Putra Malaysia, 43400 UPM Serdang, Selangor, Malaysia; snhasmahishak@gmail.com (S.N.H.I.); hafizah_kamar@upm.edu.my (N.H.A.K.); mshukuri@upm.edu.my (M.S.M.A.); adamleow@upm.edu.my (A.T.C.L.); 2 Department of Microbiology, Faculty of Biotechnology and Biomolecular Sciences, Universiti Putra Malaysia, 43400 UPM Serdang, Selangor, Malaysia; 3 Centre of Foundation Studies for Agricultural Science, Universiti Putra Malaysia, 43400 UPM Serdang, Selangor, Malaysia; 4 Department of Biochemistry, Faculty of Biotechnology and Biomolecular Sciences, Universiti Putra Malaysia, 43400 UPM Serdang, Selangor, Malaysia; 5 Department of Cell and Molecular Biology, Faculty of Biotechnology and Biomolecular Sciences, Universiti Putra Malaysia, 43400 UPM Serdang, Selangor, Malaysia; 6 Institute of Bioscience, Universiti Putra Malaysia, 43400 UPM Serdang, Selangor, Malaysia; 7 Laboratory of Halal Science Research, Halal Products Research Institute, Universiti Putra Malaysia, 43400 UPM Serdang, Selangor, Malaysia

**Keywords:** T1 lipase, hydrogen bonds, ion-pair interactions, thermostability, site-directed mutagenesis

## Abstract

A comparative structure analysis between space- and an Earth-grown T1 recombinant lipase from *Geobacillus zalihae* had shown changes in the formation of hydrogen bonds and ion-pair interactions. Using the space-grown T1 lipase validated structure having incorporated said interactions, the recombinant T1 lipase was re-engineered to determine the changes brought by these interactions to the structure and stability of lipase. To understand the effects of mutation on T1 recombinant lipase, five mutants were developed from the structure of space-grown T1 lipase and biochemically characterized. The results demonstrate an increase in melting temperature up to 77.4 °C and 76.0 °C in E226D and D43E, respectively. Moreover, the mutated lipases D43E and E226D had additional hydrogen bonds and ion-pair interactions in their structures due to the improvement of stability, as observed in a longer half-life and an increased melting temperature. The biophysical study revealed differences in β-Sheet percentage between less stable (T118N) and other mutants. As a conclusion, the comparative analysis of the tertiary structure and specific residues associated with ion-pair interactions and hydrogen bonds could be significant in revealing the thermostability of an enzyme with industrial importance.

## 1. Introduction

Lipase (triacylglycerol acylhydrolase E.C.3.1.1.3) is a class of enzyme known as serine hydrolases which function as biocatalyst for many applications such as hydrolysis, esterification, interesterification, and alcoholysis [1,2,3,4]. As a leading industrial biocatalyst, lipases can tolerate heat and to remain stable at high temperatures. The ability to tolerate heat is generally obtained from a source where catalytic reaction is naturally performed at an elevated temperature. Hence, the use of a thermostable lipase is crucial for industrial processes, because of their ability to slow the denaturation at high temperatures, which contributes to enzyme deactivation [5]. In general, thermostable enzymes are active at temperatures between 60 °C and 125 °C [6]. Moreover, thermostable lipases also are known to be resistant to the presence of chemical denaturants, detergents, organic solvents, and protein inhibitors [7].

To better understand the enzyme, 3D structure-guided protein engineering approach can be considered to be a prevalent discipline, with many researchers uncovering the folding of proteins and recognition of the protein design. The current challenge of protein engineering research is to preserve the functional properties of enzymes after modifications as the modification of protein or DNA improves the selectivity and activity of enzymes. Enzymes with high activity and stability in extreme conditions are desirable for efficient application in industry. Recent progress in 3D structure-guided protein engineering technique has permitted the improvement of enzyme functionality and scaled-up production.

Contributing factors for protein stability include hydrogen bonds, electrostatic interactions, hydrophobic interactions, disulfide bonds, and metal binding. A protein structure with good conformation such as increased rigidity, compact packing of tertiary structure, conformational strain release, increased alpha helix and reduced entropy of unfolding are determinants in achieving protein stability [6,8]. Such stabilizing factors will aid in the generation of a stable enzyme. Previously, Zhang et al. [9] successfully developed two mutants originated from *Candida antarctica* lipase B exhibiting higher activity and resistance towards irreversible thermal inactivation via an error prone polymerase chain reaction (PCR) mutagenesis. The mutants displayed an increase in half-life at 70 °C compared to the wildtype. In addition, mutant of *Stenotrophomonas maltophilia*’s lipase showing a large increase in thermostability due to the introduction of additional salt bridges caused by mutation [10]. Several protein sequences or structural features bearing structural rigidity and reduced surface hydrophobicity have been proposed to contribute to greater stability of thermophilic proteins [8]. Additionally, the re-structuring of lipase from *Bacillus pumilus* based on homology modeling study has been found to reduce hydrophobicity and increased rigidity in the protein structure, which further increased the stability of lipase [11].

T1 lipase of *Geobacillus zalihae* was isolated from palm oil mill effluent in Malaysia [12]. This lipase consists of 388 amino acids with a molecular weight of 43 kDa and showed optimum activity at 70 °C; it was active over a pH range from 6 to 11 [13]. This lipase was successfully crystallized under space and earth crystallization conditions and both structures were solved at high resolutions of 1.1 Å for space and 1.3 Å for earth [14]. An analysis of in silico and molecular dynamics performed on earth- and space-grown T1 lipase crystal structures revealed eminent differences in structure, more particular to the number of hydrogen bonds and ion-pair interactions [15]. The space-grown crystal structure exhibited extra hydrogen bonds and ion-pair interactions in its structure, which involved structure stability. The interactions between residues Gln59-Thr118, Asp43–Gln39, Glu226–Gln254, Glu250–Arg330, and Asn304–Thr306 were absent in the Earth-grown structure [15]. In another work, a mutant of T1 lipase with five mutation sites was constructed in an attempt to mimic the space-grown structure [16]. Nonetheless, the multiple mutations failed in creating non-covalent interactions between the targeted residues such as those observed in space-grown structure and which compromised the stability. In this present work, single mutants were constructed independently to determine the plausible factors contributing to the structure modification. The influences of each amino acid replacement in modulating HT1 lipase characteristics and promoting new establishment of hydrogen bond and ion-pair interaction were investigated.

## 2. Results

### 2.1. Expression and Purification of Mutated Lipases

**Figure 1:**
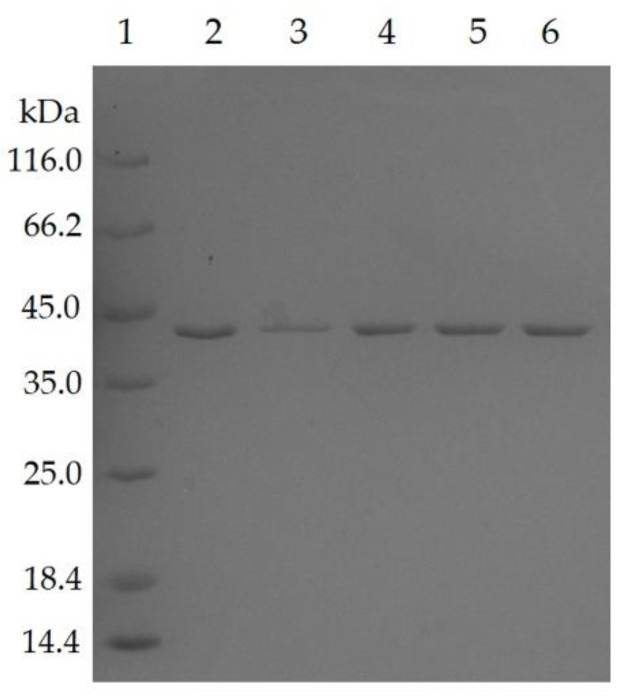
The SDS-PAGE analysis showed the purified mutated HT1 lipases after affinity chromatography. Lane 1, Marker. Lane 2, D43E. Lane 3, T118N. Lane 4, E226D. Lane 5, E250L. Lane 6, N304E. About 0.1 µg of purified mutant lipases in 20 mM sodium phosphate (pH 7.4) buffer are loaded into each well.

Five residues of T1 lipase have been identified to form additional hydrogen bond and ion-pair interaction in a space-grown crystal structure [15]. Following this, the residues were targeted to construct five single mutants, namely D43E, T118N, E226D, E250L, and N304E using a QuikChange Lightning Mutagenesis kit (Stratagene, Agilent Technologies, Santa Clara, CA, USA). The sequence alignment of mutated lipases also pointed to the presence of nucleotide change at preferred residue positions (Figure S1). All mutated lipases were subjected to purification by affinity chromatography (Ni-Sepharose High Performance, GE Healthcare, Chicago, IL, USA), in which the recovery rates of purification for mutant D43E, T118N, E226D, E250L, and N304E were 58.7%, 58.2%, 53.3%, 58.0%, and 59.9%, for respective protein yields of 456, 426, 438, 798, and 606 mg/L. All lipases were successfully purified to homogeneity, as shown by the presence of a single band at approximately 44 kDa when analyzed by SDS-polyacrylamide gel electrophoresis (Figure 1).

### 2.2. Optimum Temperature and Thermostability Study of Mutated HT1 Lipases

**Figure 2:**
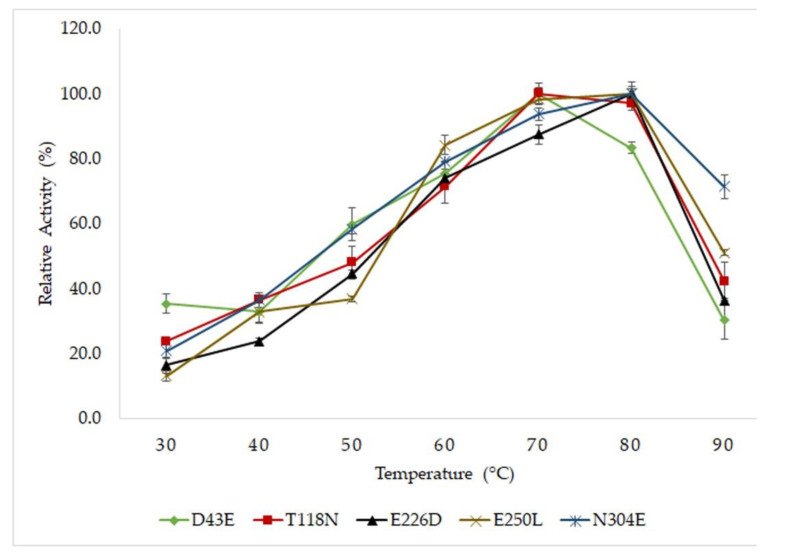
Effects of temperatures on activity of mutated HT1 lipase. The activity was determine using colorimetric assay and the optimum activity of each enzyme was recorded as 100%. The protein concentrations were standardized to 50 µg for each sample. The assays were conducted in triplicate.

All mutants displayed high activity at a temperature range of 60 to 80 °C (Figure 2). Mutants D43E and T118N demonstrated optimal activity at 70 °C, whereas mutants E226D, E250L and N304E displayed optimal activity at a higher temperature of 80 °C. The thermostability of lipases were determined by pre-incubated the protein at temperature of 60, 70 and 80 °C. The time of incubation in which the lipases lost 50% (half-life, T½) of its residual activity was determined. The half-life of mutant E226D was longer compared to other mutants, resulting in higher thermostability (Table 1). The substitution of Glu226 with Asp had showed a notable increase of half-life exhibiting a half-life values of 1680, 165 and 47 min at 60 °C, 70 °C and 80 °C, respectively. This was closely followed by mutant D43E that displayed half-lives at 900, 135 and 33 min at 60 °C, 70 °C and 80 °C, respectively. Mutation of Thr118 to Asn had the least ability to overcome high temperature by due to its shorter half-life compared to the rest of mutants, showing the half-life values of 360 min at 60 °C, 75 min at 70 °C and 10 min at 80 °C. To support the thermostability study, the melting temperature (T_m_) of the lipases were determined using circular dichroism. The melting temperature of T118N revealed a denaturation temperature of 69.3 °C. In D43E and E226D, the thermal denaturation profiles were parallel to the half-life analysis in accordance with increased in T_m_ and prolonged half-life. This result implied that mutations, in particular to D43E and E226D, had played a significant role in the stability and lipase structure at high temperatures, also leading to the improvement of catalysis.

### 2.3. Effects of pH on Mutated HT1 Lipase Activity and Stability

**Figure 3:**
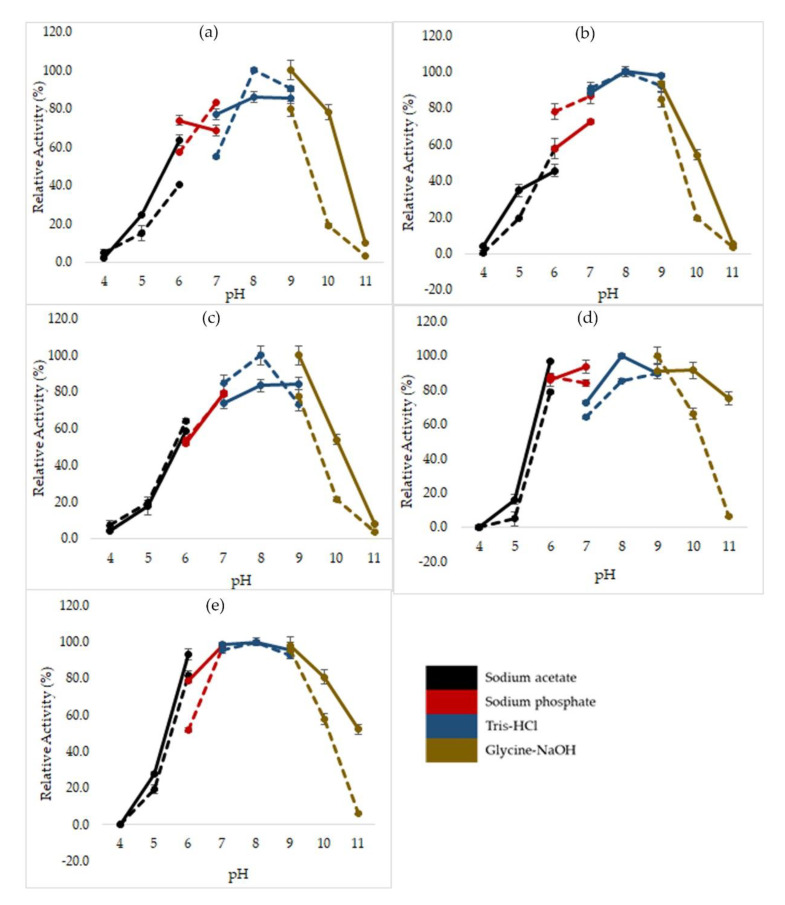
Effects of pH on activity of mutated HT1 lipases. (**a**) D43E. (**b**) T118N. (**c**) E226D. (**d**) E250L. (**e**) N304E. Dashed line represent the activity of lipase in different buffers with different pH values. Straight line represents the residual activity left after treated with buffer at different pH value for 30 min (60 °C) prior to lipase assay. The enzyme concentration was standardized to 50 µg for each sample. The assay was performed in triplicate using olive oil as substrate. The higher activity of each mutant lipase was recorded as 100%.

The determination of optimum pH of mutated HT1 lipases was performed using various buffers from pH 4 to 11 following the colorimetric method of Kwon and Rhee [17]. To study the stability of lipases in various pH range, the lipases were pre-incubated for 30 min at 60 °C in different buffers with different pH values followed by standard lipase assay at optimum condition. Most of the mutants were optimally active at Tris-HCl pH 8, except for E250L which displayed a small change in optimal pH (Figure 3). Mutant E250L possessed significantly higher activity at pH 9 by using Glycine-NaOH buffer and was increasingly stable at a broader range of pH of 6 to 11. Meanwhile, other mutants demonstrate stability at pH levels of 6 to 10. These mutants were found to display low activity at acidic pH, retaining relative activity at only 16.2 to 34.9% at pH 5.

### 2.4. Effects of Metal Ions on the Activity of Mutated HT1 Lipases

Metal ions can have a large influence on catalysis and structural modifications of enzymes. Therefore, the effects of metal ions such as Na^+^, Ca^2+^, Mg^2+^, Cu^2+^, Fe^3+^, Ni^2+^, and Zn^2+^ on lipase activity was important for measuring the functions of mutated HT1 lipases. The data analysis showed different catalytic behaviors in the tested mutants after treatment with metal ions. The results showed that calcium ions strongly stimulate the activity of the mutated HT1 lipases in particular to mutants D43E, T118N, and E250L (Table 2). The increase of activity in T118N reached up to 1.5- and 1.7-fold after treatment with 1 mM and 5 mM calcium ions, respectively. Surprisingly, the activity of T118N was enhanced even in the presence of low Fe^3+^ concentration, whereas other mutants demonstrated a decline of lipase activity with Fe^3+^. T118N had the ability to preserve 70% of remaining activity in Fe^3+^ which was not observed in other mutants under high concentration of the similar metal ion. High concentration of Ni^2+^, Fe^3+^, Cu^2+^, and Zn^2+^ could exert a negative effect on the hydrolytic activity of lipases. Unlike these metal ions, Na^+^ was found not to affect the lipase activity of tested mutants. In addition, Mg^2+^ slightly reduced the activity of mutants E226D and N304E.

### 2.5. Effect of Organic Solvents on Lipase Activity of Mutated HT1 Lipases

**Figure 4:**
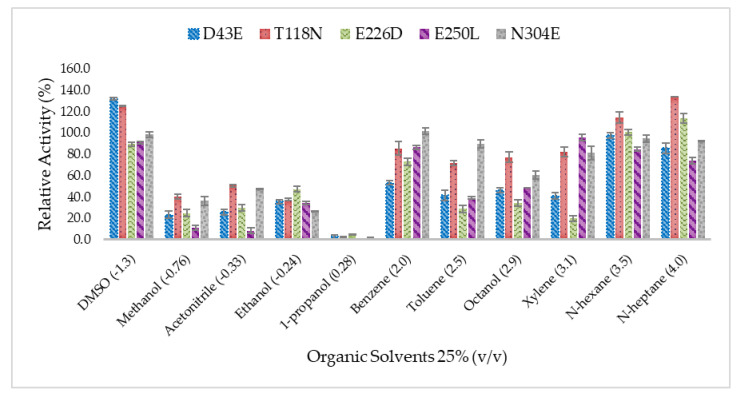
Effect of organic solvents on the catalytic activity of HT1 lipase mutants. The relative activity of each lipase was compared to the untreated lipase without organic solvents. The value of untreated enzyme for each lipase was taken as 100% and not presented in the graph.

The data analysis reveals that all mutants display different tolerances when examined in organic solvents of different type (Figure 4) The residual lipase activity in organic solvents was calculated and compared to the untreated lipase. The results showed that D43E and T118N had their relative lipase activity improved correspond to dimethyl sulfoxide (DMSO) at 129.4% and 120.6%, respectively. In addition, T118N also exhibited an increasing residual lipase activity in the presence of *n*-heptane at 130.7%. In general, water miscible solvents such as methanol, ethanol, and acetonitrile showed down-regulated lipase activity in all mutants except for DMSO. The addition of 1-propanol had caused a disruption of lipase structure which further led to lipase denaturation, whereby the ability to promote hydrolysis was reduced in 1-propanol. According to the results, T118N and N304E were highly stable lipases in organic solvents which acquire more than 80% of residual activities in DMSO, benzene, xylene, *n*-hexane, *n*-heptane, and toluene at 25% *v*/*v* concentration. Based on the well-known concept of log *P*, a low value of log *P* from organic solvent shall be the reason for the reduction of enzyme activity, whereas the non-polar (or high value log *P*) value indicates the increase of enzymatic activity. In this study, mutated HT1 lipases exhibited different dependencies on organic solvents. The lipases have been found to be considerably stable in DMSO and weaken in hydrolysis when treated with methanol, acetonitrile, ethanol, and 1-propanol. 

### 2.6. Secondary Structure Analysis of Mutant HT1 Lipases

**Figure 5:**
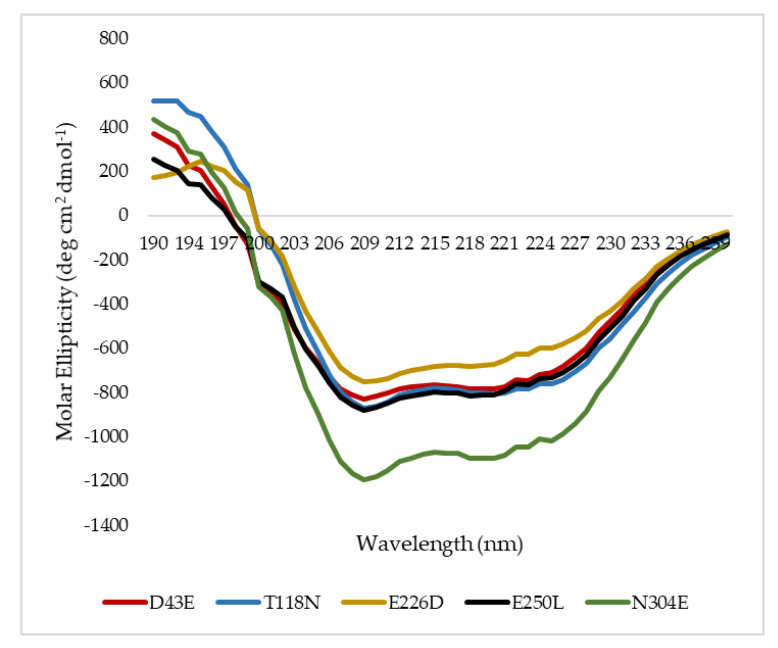
Circular dichroism (CD) spectra analysis of mutants.

To understand the influence of such mutations on the secondary structure composition of HT1 lipase, all mutated lipases were subjected to structure analysis via far-UV spectrum of circular dichroism. The superimposition of mutated HT1 lipases circular dichroism (CD) spectra at 20 °C was demonstrated (Figure 5) and the secondary structure compositions were measured using Spectra Manager^TM^ Suite Software (Japan Spectroscopic Company (JASCO), Tokyo, Japan) in which the proportion of secondary structure includes α-helix, β-sheet, turn, and random (Table 3). Mutant N304E showed to be distinctively different as compared to other mutants whereby the values were all negative between the spectra of 230 to 205 nm. However, upon analysis, there was no difference in terms of secondary structure composition between N304E mutant and other mutated HT1 lipases (Table 3). In much lesser thermostable T118N, the β-sheet structure was found to be less than the other lipases. A small decline in β-sheet structure was likely due to the changes in the distribution of intramolecular salt bridges and hydrogen bonding [18,19].

### 2.7. Structural Investigation

**Figure 6:**
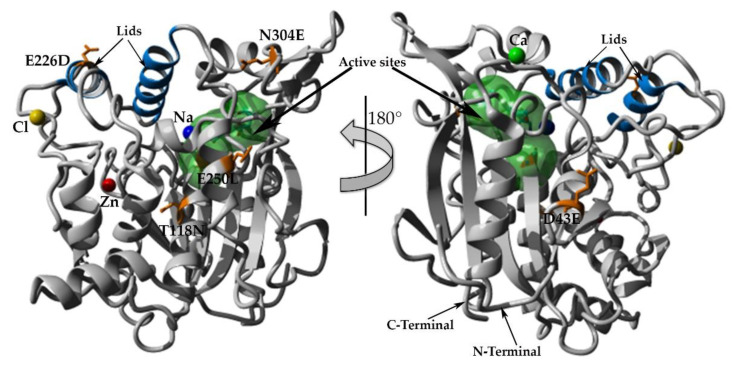
Overall structure of HT1 lipase with mutated amino acids. The figure was generated by YASARA software [20]. Blue color helical structures represent the lids. Amino acids covered with green surface represent active sites. Mutated amino acids colored in brown. Green ball represents calcium ion, yellow ball represents chloride ion, blue ball represents sodium ion, and red ball represents zinc ion.

**Figure 7:**
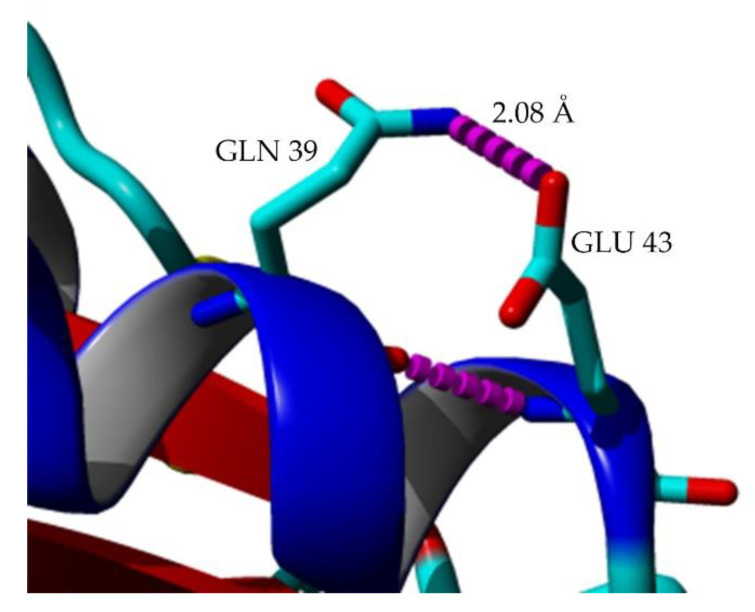
Hydrogen bond between residue Glu43 and Gln39 presented in homology model of D43E mutant structure. Hydrogen bond colored in magenta. The figure was generated using YASARA software [20].

**Figure 8:**
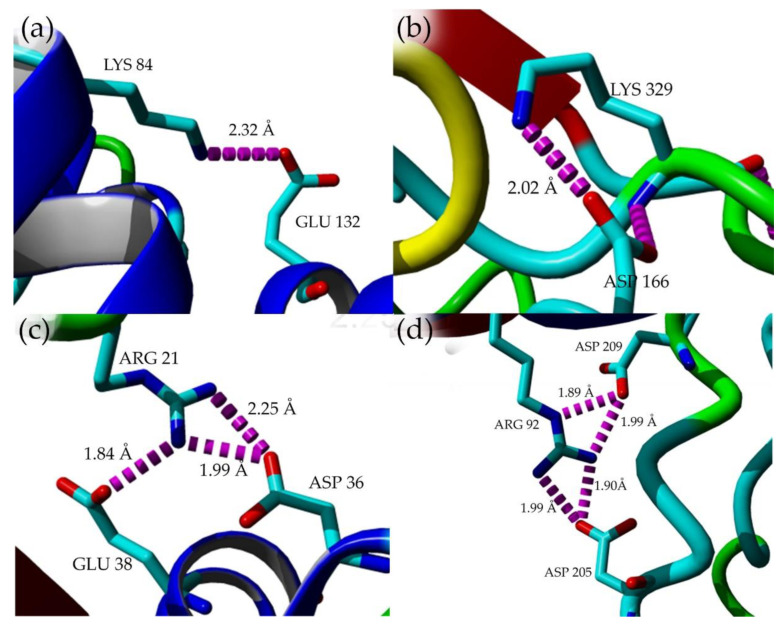
Ion-pair interaction in mutant structures. (**a**) Ion-pair interactions in D43E mutant structure between amino acid Lys84–Glu132 (**b**) Ion-pair interactions in D43E mutant structure between amino acid Lys329–Asp166 (**c**) Ion-pair network in D43E mutant structure comprising amino acids Glu38, Arg21, and Asp36. (**d**) The largest ion-pair networks in E226D structure corresponding to amino acids Asp209, Arg92, and Asp205. Ion-pair interaction colored in magenta. The figure was generated using YASARA software [20].

The model structures of mutants were obtained via homology modeling using YASARA (Yet Another Scientific Artificial Reality Application) software, and T1 lipase (PDB ID: 2DSN) as a template [20,21]. Based on the predicted structures of the mutated lipases, E226D, E250L, and N304E were localized on the surface of HT1 lipase, while T118N was reservedly located at the helix near the catalytic sites (Figure 6). D43E was found located at the first helix after N-terminal. Substitution of Asp43 to Glu resulted in a new hydrogen bond formation between Glu43 (D43E) and residue Gln39 as shown by homology model of D43E (Figure 7). The analyses on the structures showed differences in the total number of ion-pair interactions, hydrophobic interactions and aromatic interactions (Table 4). T118N demonstrated a decrease in thermostability at high temperature compared to other mutated HT1 lipases. Based on the structural analysis, T118N had low molecular interaction in its structure as opposed to D43E and E226D, which exhibited an increase in numbers of ion and aromatic interactions. Substitution of Asp43 to Glu resulted in the additional ion-pair interactions between Lys84–Glu132 and Lys329-Asp166, which was not found in other mutants’ structures (Figure 8a,b). Mutant D43E also exhibited ion-pair network interaction derived from Asp36, Arg21, and Glu38 as shown in Figure 8c, unlike other mutants’ structures. As for E226D, the mutant consists of the largest ion-pair network connected by Arg92, Asp209, and Asp205 (Figure 8d). T118N was found located in the core region of the protein and that affects the stability of the protein. Based on the analysis of T118N structure, the substitution of Thr to bulkier amino acid, Asn led to the collapse of existing interactions due to the forceful movement of neighboring amino acids. As a result, T118N negatively affected the structure by disrupting the thermostability of protein. This condition strongly indicates the influence of site mutation on the changes of molecular interactions. The number of molecular interactions such as ion-pair interactions in protein structure could lead to improvements of lipase stability against higher temperatures, as demonstrated by D43E and E226D.

## 3. Discussion

Previously, biochemical characterization of wildtype HT1 lipase has been conducted across a wide range of parameters, including temperature, pH, metal ions, organic solvents and thermostability [16]. In terms of targeted mutations, there was a shift in optimum temperature for E226D, E250L and N304E in comparison to wildtype HT1 [16]. The change in optimum temperature was also observed in Y224C and Y224P from thermophilic lipase from *Geobacillus* sp. RD-2 (lipGRD) in which the optimum activity shifted from 55 °C to 35 °C and 65 °C for mutant Y224C and Y224P, respectively [22]. In *Bacillus* sp. lipase, the substitution of Pro247 to Ser was found to decrease the temperature optima of lipase, but surprisingly also resulted in an increase of protein stability [23].

Comparison of mutated HT1 lipases with previously characterized wildtype HT1 lipase has revealed that mutants in the form of D43E, E226D, E250L and N304E have a longer half-life and higher melting temperature (Table 5). The wildtype HT1 had a half-life time of 540, 85 and 16 min at 60, 70, and 80 °C, respectively, with a melting temperature at 70.9 °C [16]. A previous study by Syal et al. [24] demonstrated that YLIPL1 and YLIP9L1Bp3 lipases exhibited better thermostability compared to their wildtype, YLIP9 lipase. Similarly, a directed evolution on *Bacillus licheniformis* lipase resulted in an increase of lipase thermostability [25]. Wu et al. [22] reported that the substitution of amino acid from Tyr224 to Pro in *Geobacillus* sp. lipase (lipGRD) had raised the half-life of lipase for 8 h as compared to wildtype (6 h) at 65 °C. In a similar study, the substitution of Tyr224 Cys had demonstrated loss of stability as compared to its wildtype. As shown in our result, amino acid substitution of Thr 118 to Asn resulted in decreasing of its stability. Lan et al. [26] had reported that substitution of Asn to Asp at position 277 could have imposed a different characteristic towards temperature. The mutant displayed an increase of lipase thermostability, and when the same Asn277 was replaced with Leu and Val, the lipase stability declined sharply. In other scenarios, when the substitution was made with Phe, the enzyme turns out to have defected. Other studies on *Geobacillus* lipases such as lipases from *Geobacillus stearothermophilus* L1, *Geobacillus* sp GD-95-10, and *Geobacillus* sp NTU, showed that lipase variants lost their thermostability due to the mutation [27,28,29]. In this study, all lipases displayed low activity in acidic pH and were totally deactivated at pH 4. According to Sharma et al. [30], low activity in acidic conditions is a normal behavior of lipase. As reported by Ali et al. [31], the *Geobacillus* lipase F16L mutant was significantly unstable at a pH below 6 and above 10. Similar trends have been shown by T118N and E226D mutants.

Protein requires metal ions for catalysis and to retain the stability of structure under extreme conditions [32,33]. Calcium ions are important in protein stability, and so the activities of some lipases from *Staphylococcus* sp. and *Geobacillus* sp. [34,35] are highly dependent on these ions. The addition of calcium increases the stability of lipases from *B. thermoamylovorans* at elevated temperature [36]. Similarly, calcium-dependent stabilization has also been reported in *Bacillus circulans* and *Staphylococcus saprophyticus* lipases [37,38]. From these results, calcium can protect enzymes from thermal denaturation and could be considered vital in maintaining the active conformation of the enzyme, be at high temperature. However, other metal ions in the form of Ni^2+^, Fe^3+^, Cu^2+^, and Zn^2+^ have demonstrated an inhibitory effect on enzyme activity. A similar finding was reported by Yamada et al. [36], in which zinc ion was shown to potentially reduce the activity of lipase from *Bacillus thermoamylovorans* (Lip501r), while in the presence of calcium ion, the lipase activity could be still enhanced. In this study, the presence of Fe^3+^ has been shown to inhibit the activity in most mutants. However, in the case of T118N, a small amount of Fe^3+^ was able to enhance the lipase activity at 129.9%, and with an increased amount of Fe^3+^ ion, enzyme activity was slightly inhibited. According to Ghori et al. [39], the increase of lipase activity in the presence of metal ions is a clear indication that those ions do not compete with the enzyme. As for inhibited lipase activity caused by metal ions, inhibition takes the form of a competitive inhibition rendering enzyme to reduced catalytic activity.

The stability of T118N and N304E have been reported to be more than 80% of residual activities when exposed to various organic solvents such as DMSO, benzene, xylene, n-hexane, *n*-heptane, and toluene. T118N was stable in the presence of organic solvents even though the thermal stability was at a decline. Lipase activity of T118N was high compared to other mutants in methanol, acetonitrile, and octanol. In general, amino acid substitutions able to induce structural changes, including the formation of a salt bridge and hydrogen bonds, improved packing of the hydrophobic core, and pI shift of side chain. As for S155L, G157R, S164K, S194R, and D209N from LST-lipase, these mutants have been reported to possess improved stability to organic solvents via structural changes [40]. In A8T, A92E, N97Q, and T245S, the stability of mutants in 80% of methanol increased due to the improvement of hydrogen bond interactions which strengthened the binding of water molecules on the surface of the enzyme. The tightly bound water molecules on the surface of the enzyme might reduce the water stripping effect in the presence of methanol which leads to enzyme stabilization. Mutant N304E, located at the loop on the surface of the protein, is considered a dynamic region of the protein. The effect on the substitution of a polar uncharged amino acid (Asn) to a negatively charged amino acid (Glu) caused in the stabilization of lipase in benzene and toluene. N304E has also been shown to have increased thermostability due to a longer half-life and improved melting temperature. Contradicting the current findings, Shokri et al. [41] reported that substitution of Asn to charged amino acid (Glu and Asp) would contribute to a decrease in enzyme stability in organic solvents, whereas substitution with Ile and Leu have been shown to increase the stability due to the improvement of hydrophobic patches. The hydrophobic patches of the enzyme surface do enhance the stability of the said enzyme in an organic solvent. Substitution of Ser with hydrophobic amino acid caused in a decline of enzyme activity in organic solvents condition that can be explained by the presence of polar side chain of Ser, due to allowing for the water solvation needed for proper folding and stability. Such replacement of Ser residue leads to a decrease of structural stability in both aqueous and organic solvents, suggesting that structural stability of the enzyme was a contributing factor to the stability in organic solvents [41]. An increase in thermostability for N304E is likely due to the increase in structural rigidity at the most active part of the enzyme due to an additional hydrogen bond that concurrently stabilized the structure in organic solvents. This hydrogen bond has the function to hold water molecules tightly on the surface of protein minimizing water stripping. As a result, amino acids play an important role in modulating lipase activity in organic solvents. Previously, by introducing polar or charged amino acid residues in between surface residues, the stability of the enzyme in organic solvents has been enhanced. A few examples are mutations of N97Q, N264Q, and D265E which are located on the enzyme surface causing the shift of *Candida antarctica* lipase stability in various organic solvents [42].

Using the structure models of HT1 mutants, differences in molecular interactions have been observed. The addition of ion-pair interactions in lipase structure has been the pillar of thermostability in lipases as indicated by mutants E226D and D43E through biochemical characterizations. According to previous molecular dynamics simulation analysis, the interaction between Glu226 and Arg230 in the Earth-grown lipase structure was found less stable compared to the same interaction in space-grown lipase structure [15]. From here, the interaction was predicted to result in enzyme stability by with Glu substituted with Asp. The proposed mutation was expected to attract other forms of interactions within the structure that would alleviate the stability of the structure at high temperatures. The increase in the number of molecular interactions and additional ion-pair interactions within the structures of D43E and E226D may contribute to the thermostability of proteins. The association of molecular interactions with thermostability has been determined previously via the introduction of additional interactions in lipase from *Geobacillus stearothermophilus* which resulted in the improvement of lipase stability [43]. Both hydrogen bond and ion-pair interaction are important structural features involved in protein folding and is an essential component to preserve the catalytic structure and to regulate the unfolding mechanism of protein structure [44]. These molecular interactions, along with other interactions such as hydrophobic interaction and aromatic interaction, may rigidify and strengthen the protein structure. Such rigidity might be useful to improve protein stability against harsh conditions such as organic solvents and high temperatures that are the main cause of enzyme denaturation. Previous studies have suggested that engineering ion-pair interactions and hydrogen bonds by amino acid substitution could improve the stability of a protein [10,26]. In this study, a decrease in the number of molecular interactions exhibited by T118N led to the destabilization of its’ structure, as well as decreased melting temperature and stability of mutant at elevated temperatures. Charbonneau and Beauregard [45] demonstrated the role of ion-pair interactions in thermostability through disruption of ion-pair interactions, in which causing destabilization of protein led to a decrease in melting temperature. In another study of a thermophilic lipase, a substitution involving Ser130 to Thr caused in a loss of a single hydrogen bond crucial to maintaining the structure of lipase. Because of the loss of hydrogen bonds, the stability of thermophilic lipase was reduced [30].

## 4. Materials and Methods

### 4.1. Site-Directed Mutagenesis

Site-directed mutagenesis was performed using the QuikChange Lightning Mutagenesis kit (Stratagene, Agilent Technologies, California, CA, USA) following the manufacturer’s protocol of modified plasmid pGEX-4T-1/His with wildtype gene of T1 lipase (pGEX-4T-1/HT1) as the template with primers containing the corresponding mutations sites (Table 6). Previously, plasmid pGEX-4T-1/His was modified by replacing a GST-tag in pGEX-4T-1 with a His-tag for the purpose of a facile purification via single-step affinity chromatography [46]. Subsequently, the purified PCR products were transformed into XL10-Gold ultracompetent cells. Plasmid extraction was carried out using MYgen plasmid mini prep kit (Gene Xpress PLT, Selangor, Malaysia). The purified plasmid was sequenced to confirm and validate on the presence of mutation sites. Positive clones of mutant were sub-transformed into expression host *Escherichia coli* strain BL21(DE3)pLysS.

### 4.2. Expression and Purification of Lipase

*E. coli* strain BL21(DE3)pLysS harboring mutated plasmid was inoculated in 10 mL of Luria Bertani (LB) broth supplemented with 100 µg/mL ampicilin and 35 µg/mL chloramphenicol. The bacteria were grown at 37 °C overnight with shaking at 150 rpm. The cultures were then transferred into 200 mL of freshly prepared similar medium until reaching an OD_600_ of 0.6–0.7 for induction which was performed by adding a 0.025 mM of isoprophyl-l-d-thiogalactopyranoside (IPTG). After 12 h of culture at 37 °C, the cells were harvested by centrifugation at the speed of 10,000× *g* for 30 min at 4 °C. The pellet was retrieved and re-suspended with binding buffer (20 mM sodium phosphate buffer pH 7.4 containing 0.5 M sodium chloride, and 5 mM imidazole). About 5 mM dithioreitol (DTT) was added prior to sonication. During purification, a High-Performance Nickel Sepharose resin was used, carefully packed into a XK 16/20 column and sufficiently equilibrated with a matching binding buffer prior to sample loading. The washing step was performed using the same buffer until no proteins or contaminants were detected. The bound protein was eluted by gradient with elution buffer (20 mM sodium phosphate, 0.5 M NaCl, pH 7.4) containing 0.5 M of imidazole. An approximate 0.1 µg of eluted proteins were loaded into the 10% sodium dodecyl sulfate polyacrylamide gel electrophoresis (SDS-PAGE) to view the purity and homogeneity of protein.

### 4.3. Lipase Assay and Protein Concentration Determination

The biochemical assay of lipase was performed using the colorimetric method according to the modified Kwon and Rhee method [17]. The substrate emulsion was prepared by emulsifying olive oil (Bertolli, Deoleo S.A., Córdoba, Spain) and buffer solution under a ratio of 1:1. During the assay, the concentration of protein or lipase used to perform assay was standardized to 5 mg/mL. In the final preparation, the assay mixture contained 10 μL of the enzyme, 990 μL of the buffer (50 mM Glycine-NaOH pH 9.0), 250 mL of substrate emulsion, and 20 μL of 20 mM calcium chloride (CaCl_2_). The mixture was incubated at 70 °C for 30 min at 200 rpm shaking. When the 30 min of incubation was completed, 1 mL of 6*N* HCl was added to end the reaction followed by the addition of 5 mL of isooctane. The reaction mixtures were vortexed and left for 30 min at room temperature to separate the oil and buffer containing oleic acid. After that, the upper layer of the reaction mix was taken and mixed with 1 mL of copper pyridine solution pH 6.1 in another vial. The activity was calculated by measuring the absorbance at 715 nm. One unit (U) is the amount of enzyme that catalyzes the reaction of one μmol of substrate per minute. Protein concentrations were calculated according to the method of Bradford [47] using the Bradford reagent (AMRESCO^®^, Avantor, Ohio, OH, USA).

### 4.4. Optimum Temperature and Thermostability Study of Mutated Lipases

The mutated lipases were tested in a broad temperature range from 30 °C to 90 °C under an agitation rate of 150 rpm using olive oil as a substrate. Each optimal enzyme activity was recorded as 100%. The thermostability of the lipase was determined by pre-incubating the enzyme solution for 36 h, 3 h and 1 h at 60 °C, 70 °C and 80 °C, respectively. Subsequently, the remaining activity of the enzyme was measured according to the standard assay. The relative activity of each sample was measured as a percentage compared to the untreated enzymes. The melting temperature (T_m_) of the lipases were determined using CD Spectropolarimeter J-815 (Japan Spectroscopic Company (JASCO), Tokyo, Japan). The concentration of each enzyme was fixed at 1 mg/mL dissolved in 5 mM Tris-HCl buffer with pH 8. Before each run, one cm optical path length was used and the warming period was set from 30 °C to 90 °C, with a heating rate of 1 °C/min at 222 nm of wavelength. The melting temperature analysis was based on the protocol of Greenfield [48]. The denaturation temperature (T_m_) was defined as the stage at which 50% of the protein sample was denatured.

### 4.5. Effect of pH on Lipase Activity and Stability

The optimum pH of each lipase was determined by measuring the hydrolytic activity at various pH range (50 mM sodium acetate buffer, pH 4.0–6.0; 50 mM sodium phosphate buffer, pH 6.0–7.0; 50 mM Tris-HCl, pH 7.0–9.0; 50 mM Glycine-NaOH buffer, pH 9.0–11.0). The pH stability was evaluated by pre-incubating the enzymes in different pH buffers for 30 min at 60 °C following the standard lipase assay.

### 4.6. Effect of Various Metal Ions on Lipase

The stability of the lipase was evaluated in response to the presence of metal ions by measuring the lipase activity after separate pre-incubation with calcium chloride dehydrate (CaCl_2_·2H_2_O), magnesium chloride (MgCl_2_), sodium chloride (NaCl), nickel(II) sulfate hexahydrate (NiSO_4_·6H2O), zinc chloride (ZnCl_2_), iron(III) chloride (FeCl_3_), and copper(II) chloride (CuCl_2_). The samples were pre-incubated in 1 mM and 5 mM of metal ions at 60 °C for 30 min following the standard lipase assay. The relative activity of the enzymes was measured and calculated to determine any differences with the untreated enzymes (100%).

### 4.7. Effect of Organic Solvent on Lipase Stability

Purified lipase was pre-incubated with organic solvents (25% *v/v*) for 30 min at 60 °C under 150 rpm of shaking. Solvents were selected based on log *P* values (values in parenthesis) and boiling point that is greater than 60 °C, wherein such organic solvents include DMSO (−1.4), methanol (−0.7), acetonitrile (−0.4), ethanol (−0.2), 1-propanol (0.3), benzene (2.0), toluene (2.5), octanol (2.9), xylene (3.1), n-hexane (3.5), n-heptane (4.0). The residual activity was measured using standard lipase assay at 70 °C using olive oil (emulsified, 1:1 *v/v* in buffer) as a substrate. The stability was expressed as the remaining activity relative to the control (without solvent).

### 4.8. Circular Dichroism Studies

The secondary structures of lipases were determined using CD Spectropolarimeter J-815 (Japan Spectroscopic Company, Tokyo, Japan). The purified lipases at 0.5 mg/mL were equilibrated in 5 mM Tris-HCl, pH 8.0. The protein sample was measured in the far-UV wavelength of 190 to 240 nm using 1 mm path cell length at 20 °C. The compositions of the secondary structures were determined using Spectra Manager^TM^ Suite Software (Japan Spectroscopic Company, Tokyo, Japan). During each measurement, a spectral reading from the corresponding buffer (5 mM Tris-HCl (pH 8.0) was used and subtracted from the CD signal of the sample. Secondary structures were identified in a form of α-helix, β-sheet, turn, and random. The derivation of secondary structure analysis was based on the protocol of Greenfield [49].

### 4.9. Homology Modeling and Structural Analysis

The putative mutations were identified based on the information of in silico analyses and molecular dynamics simulation of the space- and Earth-grown T1 lipase crystal structure [17]. The comparison of both structures revealed additional hydrogen bonds and ion-pair interactions in space-grown structure at amino acid position Asp43, Thr118, Glu226, Glu250, and Asn304. Crystal structure of T1 lipase ((PDB ID: 2DSN) was used as template for structure prediction of mutated lipases. The structures of mutants were modeled using YASARA software [20]. The analysis of molecular interactions (hydrogen bonds, ion-pair interactions, hydrophobic interactions, and aromatic interactions) on model structures of mutants were executed using module available in YASARA software with default parameters. 

### 4.10. Statistical Analysis

Each experiment was performed in triplicate. Statistical analyses were performed using one-way analysis of variance (ANOVA) of variance in Microsoft Excel using default parameters. The result was considered significantly different at *P* < 0.05.

## 5. Conclusions

In this study, the thermostability of mutant lipases was improved via mutagenesis. The lipases were successfully purified to homogeneity via single-step affinity chromatography. Biochemical and biophysical studies of mutants conclude that mutants D43E, E226D, E250L, and N304E successfully enhanced lipase thermostability. The structure and function relationship of the mutations in particular to D43E and E226D indicated that the presence of additional ionic interactions could be associated with the single-point mutations. Thus, such residues harboring additional ionic interactions could play important roles in the conformational changes of lipase structure and thermostability. The mutant T118N, located near the catalytic site, affecting several stabilizing interactions and lost thermostability. However, it was highly stable in benzene, xylene, and toluene, meanwhile, exhibited increment in activity in DMSO, *n*-hexane, and *n*-heptane. Therefore, our findings provide an understanding of the structures and functions of lipases, which have an important impact on biotechnological and industrial applications.

## Figures and Tables

**Table 1:** Thermostability study of mutants HT1 lipase.

Lipases	T½ (Min)			Thermal Denaturation(*T_m_*) (°C)
	60 °C	70 °C	80 °C	
D43E	900	135	33	76.0 ± 1.2
T118N	360	75	10	69.3 ± 0.3
E226D	1680	165	47	77.4 ± 2.6
E250L	540	115	17	71.7 ± 0.1
N304E	600	120	30	74.3 ± 0.2

**Table 2:** Effect of metal ions on the lipase activity of mutated HT1 lipases. The relative activity of each lipase was compared to the untreated lipase without metal ions. The value of untreated enzyme for each mutant HT1 lipase was taken as 100% and not presented in the table.

Lipase	Concentration (mM)	Na^+^	Ca^2+^	Mg^2+^	Fe^3+^	Ni^2+^	Cu^2+^	Zn^2+^
D43E	1	114.3 ± 3.5	123.2 ± 2.9	109.5 ± 4.8	70.6 ± 0.5	102.9 ± 2.2	22.5 ± 0.5	5.5 ± 0.1
	5	84.4 ± 0.4	156.4 ± 3.0	111.3 ± 1.8	9.4 ± 0.1	19.2 ± 3.6	6.3 ± 0.4	4.7 ± 0.1
T118N	1	100.6 ± 4.6	154.0 ± 5.1	115.9 ± 0.3	129.9 ± 4.8	32.8 ± 1.1	66.6 ± 3.0	5.7 ± 1.0
	5	91.7 ± 3.5	172.8 ± 2.3	111.4 ± 1.9	70.8 ± 2.5	10.5 ± 0.4	28.9 ± 1.2	5.3 ± 0.4
E226D	1	112.7 ± 4.5	110.1 ± 1.8	74.6 ± 4.9	72.3 ± 1.4	44.7 ± 4.3	18.6 ± 2.6	8.8 ± 1.5
	5	100.2 ± 5.1	119.7 ± 3.1	77.8 ± 4.0	5.6 ± 0.4	12.5 ± 1.9	3.5 ± 1.0	2.2 ± 0.6
E250L	1	103.3 ± 5.3	150.0 ± 4.6	97.5 ± 2.7	20.0 ± 2.2	11.7 ± 2.7	64.6 ± 1.8	2.7 ± 1.3
	5	106.6 ± 4.2	156.0 ± 4.2	101.4 ± 5.9	2.0 ± 0.9	0.8 ± 2.4	7.0 ± 0.5	11.2 ± 0.8
N304E	1	91.8 ± 4.0	101.1 ± 4.9	88.5 ± 4.7	54.1 ± 1.2	46.3 ± 4.3	27.4 ± 3.5	47.7 ± 6.7
	5	96.9 ± 3.7	106.8 ± 3.3	61.0 ± 3.9	8.1 ± 2.1	40.7 ± 6.1	7.9 ± 4.0	9.8 ± 5.4

**Table 3:** Estimated secondary structure composition (%) of mutated HT1 lipases.

	D43E	T118N	E226D	E250L	N304E
α-Helix	20.80	29.10	24.40	21.60	24.40
β-Sheet	23.60	9.80	17.90	19.80	14.20
Turn	18.10	24.30	23.40	21.10	23.30
Random	37.50	36.80	34.30	37.50	38.10

**Table 4:** Number of molecular interactions in single mutant modeled structures calculated using module available in YASARA software [20].

Lipases	Hydrogen Bond	Ion-Pair Interaction	Hydrophobic Interaction (Sidechain)	Aromatic Interaction
D43E	338	30	674	41
T118N	304	7	653	37
E226D	331	28	662	39
E250L	337	25	680	41
N304E	342	22	681	42

**Table 5:** Characteristics of wt-HT1 lipase [16].

Thermostability (T_1/2_) (Min)			T_m_ (°C)	Relative Activity in DMSO (%)	Relative Activity in Ca^2+^ (%)	
60 °C	70 °C	80 °C			1 mM	5 mM
540	85	16	70.9 ± 0.1	105.8 ± 4.2	122.0 ± 1.7	123.0 ± 4.3

**Table 6:** Mutagenic primers for the construction of HT1 mutants. The sequence in bold is the mutated sites.

Primer	Nucleotide Sequence (5′–3′)
D43E	Forward 5′3′: caatggctgaacga**g**aacggttatcgaac Reverse 5′3′: gttcgataaccgtt**c**tcgttcagccattg
T118N	Forward 5′3′: caaggggggcaga**ac**gcccgcatgcttg Reverse 5′3′: caagcatgcgggc**gt**tctgccccccttg
E226D	Forward 5′3′: gaccattattttga**t**cggctcaagcgctc Reverse 5′3′: gagcgcttgagccg**a**tcaaaataatggtc
E250L	Forward 5′3′: gatttatccgtttccggagct**tt**gaagttgaatcaatggtccac Reverse 5′3′: gtggaccattgattcaacttc**aa**agctccggaaacggataaatc
N304E	Forward 5′3′: ggttcgtaccgc**g**a**g**ccgacgctcggc Reverse 5′3′: gccgagcgtcgg**c**t**c**gcggtacgaacc

## Supplementary Materials

The following are available online, Figure S1: Partial sequence alignments of mutants D43E, T118N, E226D, E250L and N304E with wild-type HT1 sequence. (a) Asp (GAC) to Glu (GAG). (b) Thr (ACG) to Asn (AAC). (c) Glu (GAA) to Asp (GAT). (d) Glu (GAG) to Leu (TTG). (e) Asn (AAT) to Glu (GAG). The multiple sequences alignment was generated from https://www.ebi.ac.uk/Tools/msa/clustalo/. The mutated sequences are marked in red boxes.
