# Supplementary material for: Ion-Pair Interaction and Hydrogen Bonds as Main Features of Protein Thermostability in Mutated T1 Recombinant Lipase Originating from Geobacillus zalihae
Source: Molecules. 2020 Jul 28;25(15):3430. doi: 10.3390/molecules25153430 (PMC7435748; doi:10.3390/molecules25153430)
Supplement: Supplementary file 1 [file molecules-25-03430-s001.pdf]

molecules

Type of the Paper (Article, Review, Communication, etc.)

Ion interaction and hydrogen bonds as main features of protein thermostability in mutated T1 recombinant lipase originated from *Geobacillus zalihae*

Siti Nor Hasmah Ishak[1,2], Nor Hafizah Ahmad Kamarudin [1,3], Mohd Shukuri Mohamad Ali[1,4], Adam Thean Chor Leow[1,5,6], and Raja Noor Zaliha Raja Abd. Rahman[1,2,6,7,*]

[1] Enzyme and Microbial Technology Research Centre, Faculty of Biotechnology and Biomolecular Sciences, Universiti Putra Malaysia, 43400 UPM Serdang, Selangor, Malaysia; snhasmahishak@gmail.com; hafizah_kamar@upm.edu.my (N.H.A.K.); adamleow@upm.edu.my (T.C.L.); mshukuri@upm.edu.my (M.S.M.A.).

[2] Department of Microbiology, Faculty of Biotechnology and Biomolecular Sciences, Universiti Putra Malaysia, 43400 UPM Serdang, Selangor, Malaysia.

[3] Centre of Foundation Studies for Agricultural Science, Universiti Putra Malaysia, 43400 UPM Serdang, Selangor, Malaysia

[4] Department of Biochemistry, Faculty of Biotechnology and Biomolecular Sciences, Universiti Putra Malaysia, 43400 UPM Serdang, Selangor, Malaysia.

[5] Department of Cell and Molecular Biology, Faculty of Biotechnology and Biomolecular Sciences, Universiti Putra Malaysia, 43400 UPM Serdang, Selangor, Malaysia.

[6] Institute of Bioscience, Universiti Putra Malaysia, 43400 UPM Serdang, Selangor, Malaysia.

[7] Laboratory of Halal Science Research, Halal Products Research Institute, Universiti Putra Malaysia, 43400 UPM Serdang, Selangor, Malaysia

* Correspondence: rnzaliha@upm.edu.my

Received: date; Accepted: date; Published: date

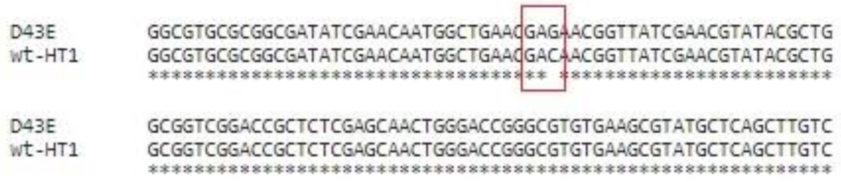

(a)

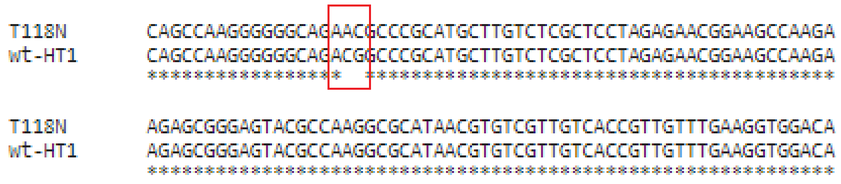

(b)

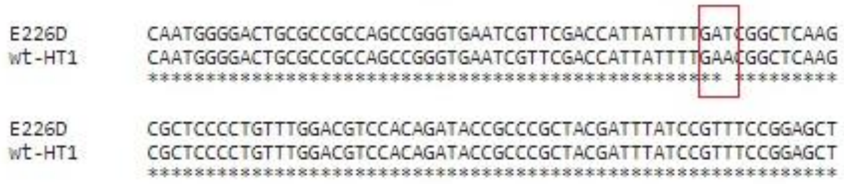

(c)

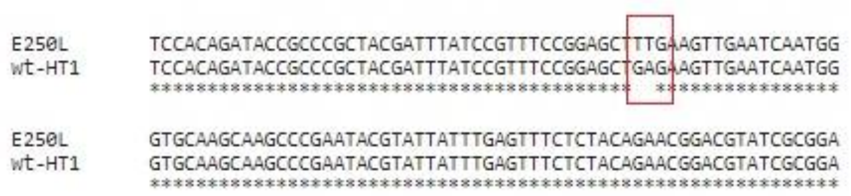

(d)

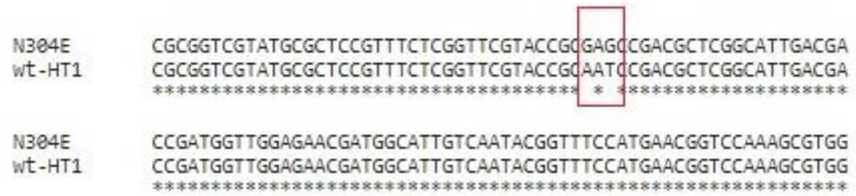

(e)

Figure S1. Partial sequence alignments of mutants D43E, T118N, E226D, E250L and N304E with wild-type HT1 sequence. (a) Asp (GAC) to Glu (GAG). (b) Thr (ACG) to Asn (AAC). (c) Glu (GAA) to Asp (GAT). (d) Glu (GAG) to Leu (TTG). (e) Asn (AAT) to Glu (GAG). The multiple sequences alignment was generated from https://www.ebi.ac.uk/Tools/msa/clustalo/. The mutated sequences are marked in red boxes.

© 2020 by the authors. Submitted for possible open access publication under the terms and conditions of the Creative Commons Attribution (CC BY) license (http://creativecommons.org/licenses/by/4.0/).
